# Differential effects of pollution on adult and recruits of a canopy-forming alga: implications for population viability under low pollutant levels

**DOI:** 10.1038/s41598-020-73990-5

**Published:** 2020-10-20

**Authors:** Sònia de Caralt, Jana Verdura, Alba Vergés, Enric Ballesteros, Emma Cebrian

**Affiliations:** 1 GRMAR, Institute of Aquatic Ecology (IEA), University of Girona (UdG), c/ Maria Aurèlia Capmany 69, 17003 Girona, Spain; 2 Centre for Advanced Studies of Blanes (CEAB), Spanish National Research Council (CSIC), c/ d’accés a la Cala St Francesc 14, 17300 Blanes, Spain

**Keywords:** Marine biology, Environmental impact, Conservation biology

## Abstract

Marine macroalgal forests are highly productive and iconic ecosystems, which are seriously threatened by number of factors such as habitat destruction, overgrazing, ocean warming, and pollution. The effect of chronic, but low levels of pollutants on the long-term survival of the canopy-forming algae is not well understood. Here we test the effects of low concentrations (found in good quality water-bodies) of nitrates, heavy metals copper (Cu) and lead (Pb), and herbicides (glyphosate) on both adults and recruits of *Carpodesmia crinita*, a Mediterranean canopy forming macroalga. We show that although adult biomass, height and photosynthetic yield remain almost unaffected in all the assays, low Cu levels of 30 µg/L completely suppress adult fertility. In addition, all the assays have a strong and negative impact on the survival and growth of recruits; in particular, glyphosate concentrations above 1 µg/L almost totally inhibit their survival. These results suggest that the long-term viability of *C. crinita* may be severely compromised by low pollutant levels that are not affecting adult specimens. Our results provide important data for a better understanding of the present-day threats to marine canopy-forming macroalgae and for the design of future management actions aimed at preserving macroalgal forests.

## Introduction

Anthropogenic pressures are affecting biological and ecological ecosystems worldwide. Changes in species phenology and geographical distribution, as well as abrupt habitat shifts are well documented^1–5^. Macroalgal forests formed by species of the orders Fucales and Laminariales are structurally and functionally complex ecosystems^6–11^. These highly productive and iconic ecosystems are declining in some areas due to the accumulation of anthropogenic impacts, such as those related to intensive land use, pollution, warming or invasive species^12–22^. The loss of macroalgal forests also implies a loss or impoverishment of ecosystem services such as nutrient cycling, food production, nursery habitat provision, erosion reduction, and control of water quality^13^. Globally, deterioration of these complex habitats is often associated with a replacement of canopy-forming algae by ephemeral and structurally simpler macroalgae, affecting the status and functioning of these ecosystems^13,23^.

Marine ecosystem shifts can be gradual and can take long periods of time to become evident^24–26^ hiding the possible causes that provoke their declines. Progressive decline of macroalgal forests can be the result of several subtle and chronic causes over the canopy-forming algae, but with drastic consequences in the long term^14^. There is a current lack of knowledge on the subtle anthropogenic stressors affecting the permanence of macroalgal forests, which might be behind the local declines of these ecosystems. However, policy and management initiatives that seek to conserve or recover lost habitats, require the capacity to anticipate and suppress the mechanisms that drive such losses^27^.

Several species of the order Fucales, which are amongst the most important canopy-forming brown algae in shallow temperate rocky bottoms^28,29^, have experienced severe declines in the North-Western Mediterranean due, amongst other causes, to pollution and eutrophication^26,30–37^ that began in the 1970s and increased steadily until the 1990s^38,39^. At the same time, biocides used in antifouling paints, for recreational boats, containing toxic compounds gained popularity in the 1970s^40^. It was not until the implementation of the EU Water Framework Directive (2008/56/EC) that the amount of pollution steadily decreased in EU Mediterranean countries due to the construction of water treatment plants^35,41^.

Some studies provide unequivocal evidences that pollution drastically affects the persistence of fucoid forests in the Mediterranean^42,43^ and it has been proved that these forests can come back, if restored, once the pollution levels have decreased and pollutants are not critical for fucoid survival^44^. However, we still need to understand the effects of pollutants at all the different macroalgae life stages. Organisms in early stages of development may have different requirements and environmental needs to those in adult stages. In fact, recruits may be more sensitive to stressors than adults, as it has already been reported for several species of *Fucus*, which in their early stages showed higher mortality, ultrastructural changes and growth retardation compared to adults when exposed to heavy metals^45–48^. The number and growth rate of recruits is related to the number of successful adult individuals^49^, which means that sublethal effects of pollutants on recruits may be affecting the long-term viability of populations. Thus, ascertaining the lethal and sublethal effects of pollution in both adults and recruits will help to provide a full understanding of population replenishment and persistence in specific environmental conditions.

Although it is difficult to disentangle the effects on the species and habitats of the different pollutants that are being thrown in Mediterranean coastal waters, here we have selected three different kinds of pollutants that by themselves are known to affect macroalgae: nitrates as a proxy for nutrient pollution^36,42,43^, copper (Cu) and lead (Pb) as representatives of pollution by heavy metals^44^, and glyphosate as a persistent organic pollutant that is one of the most widely used herbicides^50^ although is not generally considered in most marine monitoring programs^51^.

This study aims to evaluate the effects of low pollutant concentrations (nitrates, Cu, Pb and glyphosate) currently reported in non-contaminated areas of the northwestern Mediterranean Sea (i.e., continuous low/moderate concentrations of pollutants) on populations of adult and juvenile of *Carpodesmia crinita* (Duby) Orellana and Sansón (syn. *Cystoseira crinita* Duby)^52^, a canopy-forming alga that makes important shallow water macroalgal forests all across the Mediterranean^53^. Adult survival, growth, fertility and photosynthetic activity, as well as recruit density and size, were monitored from June to December 2018 under different pollution conditions. The aim was to gather information on the subtle and non-visible threats affecting the marine canopy-forming macroalgae from the northwestern Mediterranean. Such information is essential for establishing successful conservation action to preserve and restore marine forests.

## Materials and methods

### Target species and collection

*Carpodesmia crinita* (Duby) Orellana and Sansón (syn. *Cystoseira crinita* Duby)^52^ is a Mediterranean endemic species that can create dense populations in shallow and sheltered rocky shores (mainly between 0 and 1 m depth)^18,53–56^. In recent decades, *C. crinita* has experienced severe declines and even local extinctions in different areas of Spain, France, Italy, Croatia, Montenegro and Greece^31,33,34,57–61^. Although several stressors have been attributed to its demise, only Sales et al. (2011) have reported pollution as being a factor in the decline of populations of *C. crinita* and other Fucales in a Mediterranean bay.

For our study, *Carpodesmia crinita* samples were collected by a snorkeler in June 2018 from a unique population in the Natura 2000 site of Castell-Cap Roig, Cala Estreta (41°86′62.1″N, 3°17′50.7″E). Specimens, similar in shape, length and phenological state, were carefully removed from the rock while preserving their attaching disc to ensure survival during the experiment and transported immediately to the Laboratory for Experimentation with Living Organisms (LEOV) located in the Center for Advanced Studies (CEAB-CSIC) in Blanes, Spain. Once in the laboratory, epiphytes and sediment were carefully removed and specimens were placed in aquaria with ambient seawater at 18 °C and natural light levels for a one week acclimatization period.

### Treatments

Six specific treatments were applied as follows: 4 µM nitrate (nutrients); 30 µg/L copper; (heavy metal), 10 µg/L lead (heavy metal); and three different concentrations of glyphosate (herbicide), at 0.5 µg/L, 1 µg/L and 10 µg/L. The water in the tanks was renewed once a week to maintain the selected concentrations throughout the experiment. These particular concentrations were selected because they are frequently found in Mediterranean coastal waters. Although nitrate concentrations in open Mediterranean Sea waters range from 0.03 to 4.59 µM^62^, the average annual concentrations in coastal areas close to the sampling site are usually below 1 µM^42,43,54^. Nevertheless, slightly polluted areas can reach values close to 10 µM^36,43^. The concentrations of heavy metals used in Cu and Pb treatments were just above the thresholds used by the European Union to categorize a water mass as being of good environmental quality (< 25 µg/L for Cu and < 7.2 µg/L for Pb; Directive 2008/105/CE). Glyphosate has been detected at concentrations around 1 µg/L in unpolluted coastal waters^51,63,64^.

### Experimental set up

**Figure 1:**
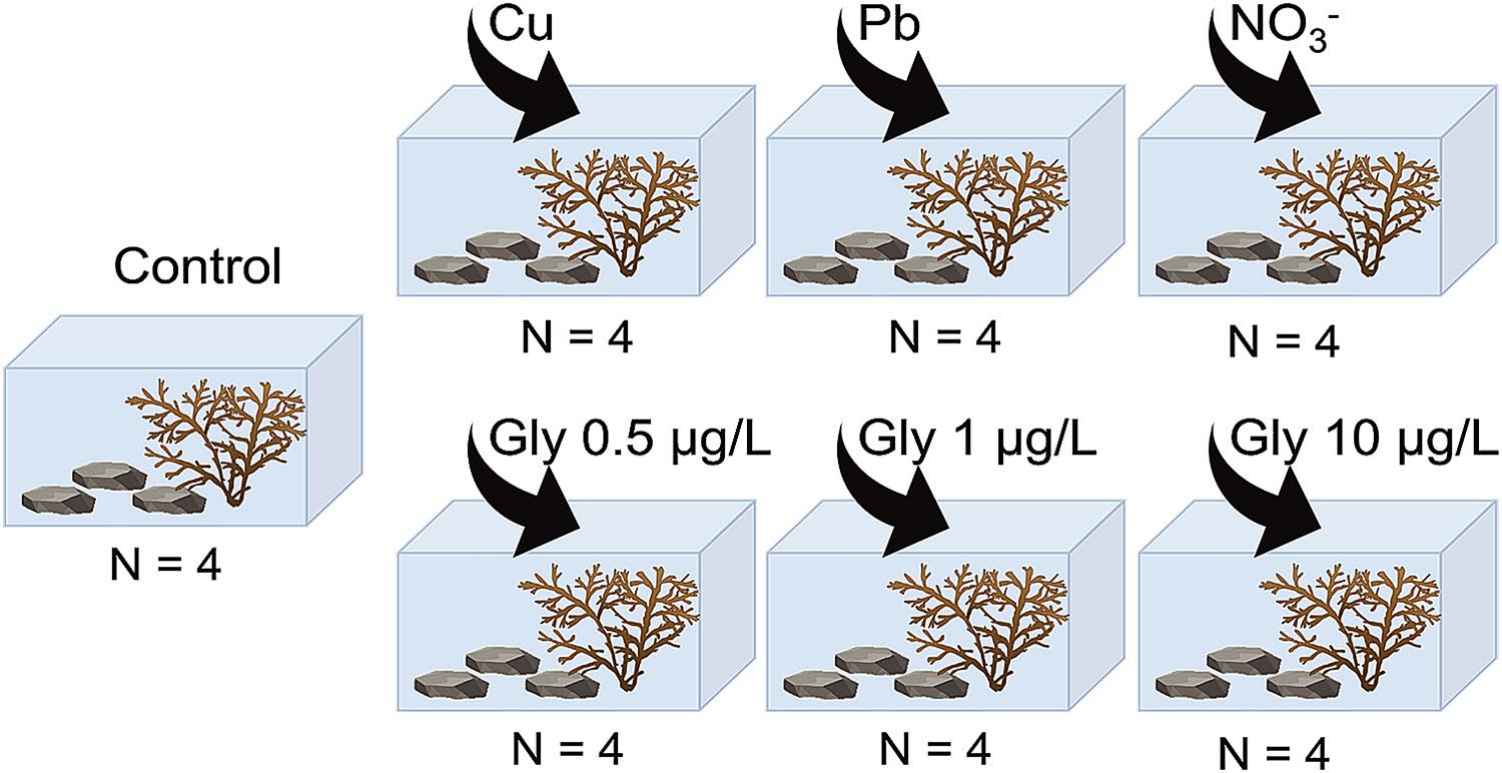
Experimental setup. Four replicate tanks (20 L) were used for each treatment (control without pollutant, 4 µM nitrate, 30 µg/L copper, 10 µg/L lead, and glyphosate at 0.5 µg/L, 1 µg/L and 10 µg/L). In each tank, one adult individual of *Carpodesmia crinita* and three stones (to provide substrata for the settlement of the zygotes) were placed. All tanks were maintained at 18 °C for 6 months, continuously aerated by air pumps, with a natural light regime and with a constant flow of natural seawater in a closed circuit.

After acclimatization, adult individuals of *C. crinita* were placed in each of the six treatments and a control (with filtered and sterilized ambient seawater), using four replicate 20 L tanks per treatment (Fig. 1). One adult individual and three large (approx. 100 cm^2^) flat stones, with even surfaces, were placed in each tank following methodology previously described to obtain recruits in experimental tanks^65^. The stones, located at the bottom of the tanks, provided adequate substrata for the settlement of zygotes, which came from fertile adult specimens located in the same tank. Recruits were visible to the naked eye after 1 month. The seawater was continuously aerated by air pumps (Hailea ACO-5504), maintained at a constant temperature of 18 °C (Hailea Chiller HC 500 A) in a natural light regime and with a constant flow of natural seawater (pump Jet NJ 3000) in a closed-water circuit, being completely replaced once a week.

### Measurements

The effects of the different pollutants on *C. crinita* was evaluated by measuring several variables both in adults and recruits. Biomass, total height and effective quantum yield (F_v_/F_m_) of photosystem II (PSII) were measured monthly in adult specimens.

Changes in wet weight and height were measured for each specimen at each sampling time. Specimens were dried with absorbent paper before being weighed in mg. The height of each adult individual was determined by measuring the length (in mm) of the longest perennial axis using a ruler. Weight and height rates (WR_t_ and HR_t_) were estimated as the change in weight and height at a given time (W_t_ and H_t_) relative to the initial measures (initial weight W_i_ and initial height H_i_) as:

$${\text{WR}}_{{\text{t}}} = \left( {{\text{W}}_{{\text{t}}} {-}{\text{W}}_{{\text{i}}} } \right)/{\text{W}}_{{\text{i}}} \;{\text{and}}\;{\text{HR}}_{{\text{t}}} = \left( {{\text{H}}_{{\text{t}}} - {\text{H}}_{{\text{i}}} } \right)/{\text{H}}_{{\text{i}}} .$$

Effective quantum yield was used as an indicator of PSII performance to assess photosynthetic efficiency. Macroalgal fronds were incubated in the dark for 15 min after which F_v_/F_m_ measurements were estimated by applying a saturation pulse using a Pulse Amplitude Modulated Fluorometry (Diving-PAM Underwater Fluorometer, Waltz, Germany) with an absorption coefficient of the leaves of 0.65, a measuring light intensity (meas-int) of 10 and an out-gain of 10 for the electronic signal gain (amplification factor). Measuring F_v_/F_m_ following a period of dark adaptation is a common technique for measuring stress in plants^66^.

A fecundity level (FL) was assigned by categorizing individuals from 0 to 4 at the beginning and at the end of the experiment: 0 (sterile individuals), 1 (less than 25% of terminal branches fertile), 2 (between 25 and 50% of terminal branches fertile), 3 (between 51 and 75% of terminal branches fertile) and 4 (more than 76% of terminal branches fertile). A fecundity index (FI) was calculated for each specimen as follows: FI = FL_f_ – FL_i_ , where FL_i_ is the initial fertility level and FL_f_ is the final fertility level.

For recruits, we only took density and mean height measures at months 3 and 6 to avoid any harm to the recruits from manipulation. The stones harboring the recruits were taken out of the water and the mean density was measured by counting the number of recruits per cm^2^ (N = 30 random samples per treatment) under the binocular microscope at 10x. The mean height of the recruits was obtained by measuring individual lengths (N = 60, random samples per treatment) under the binocular microscope using an ocular micrometer.

### Statistical analyses

Changes in wet weight, and height, photosynthetic efficiency, and fecundity index for adults and density and height for recruits, were selected as response variables. Different models were fitted to analyze the effect of the pollutants on each variable. Wet weight and height variation in adults, computed as a percentage of the initial state, were analyzed using Linear Models with Random Effects (LMER). Treatment (pollutants, plus the control; 7 levels) was used as a fixed factor, while specimen identity and time (6 levels) were used as random factors, in order to account for the lack of independence between observations repeated at different times. Generalized Linear Mixed Models (GLMM), with a quasi-Poisson error distribution and a logit link function were used to test the effects of pollutants on the Effective Quantum Yield, with treatment as a fixed factor and specimen identity and time as random. LMER and GLMM models incorporate random effects and therefore can cope with repeated measures over time^67^. Finally, the fecundity index was analyzed by means of a Linear Model (LM), with treatment as a fixed factor.

A Generalized Linear Model (GLM) with a Poisson error distribution and a logit link function, with pollutants (7 levels) and time (2 levels) as fixed factors, were used to test the effects of pollutants on recruit density. The recruit’ height data were transformed (natural log) and analyzed using a LM, with pollutants and time, once again, as fixed factors.

For all the fitted models, we applied a Type II Wald χ^2^ test to determine the effect of the fixed factors. For those models in which the effect of the fixed factors was significant, a pairwise post hoc Tukey’s Test was applied to test for differences between the different levels of the factors. All analyses were performed by statistical software R (R Development Core Team, 2014). Models were fitted using the functions “glmer”, “lmer”, “lm” and “glmmPQL” from the lme4 package^68^ and MASS package^69^. The Wald χ2 test was performed using the “ANOVA” function from the car package^70^. For multiple comparisons, we applied the Tukey test using the “lsmeans” function from the lsmeans package^71^.

## Results

### Adult responses

**Figure 2:**
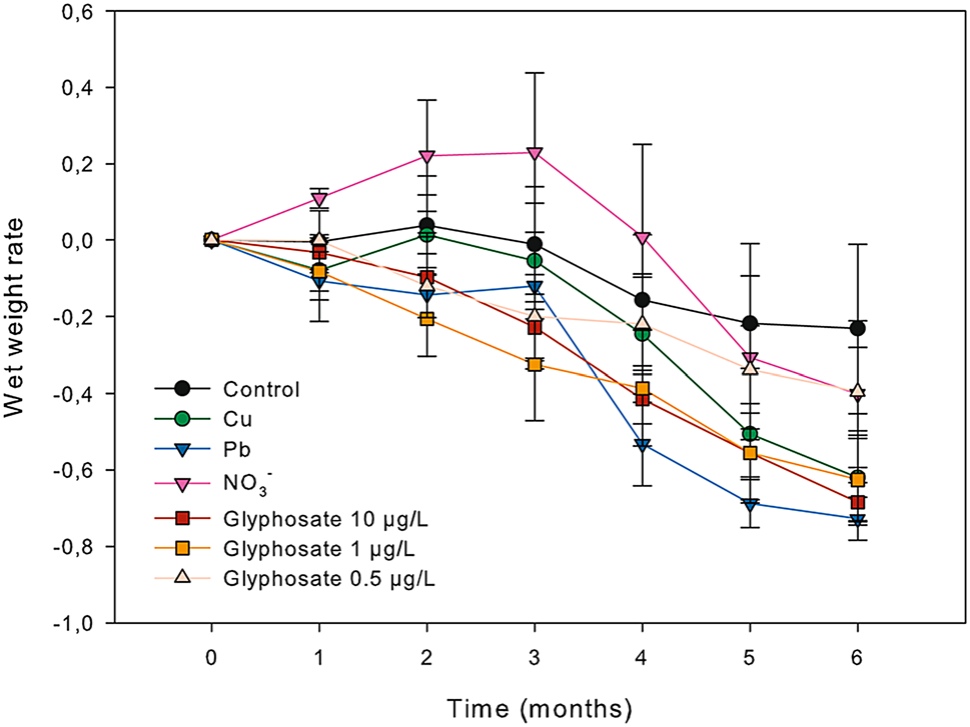
Mean wet weight variation of adult individuals of *Carpodesmia crinita* under control and the different pollutant conditions over a period of 6 months. Weight variation is given in parts per unit. Vertical bars represent standard errors.

Change in wet weight (ww) over time for each treatment of *C. crinita* is shown in Fig. 2. Wet weight was similar among treatments (χ^2^_6_ = 7.792, *p* = *0.253*; supplementary Table S1) and while weight reduction always ranged from 25 to 65% by the end of the experiment, the rate of reduction depended on the treatment. Although specimens subjected to nitrate treatment initially increased in weight, they sharply decreased its weight after the 4th month, finally reducing its weight by 40% at the end of the experiment.

**Figure 3:**
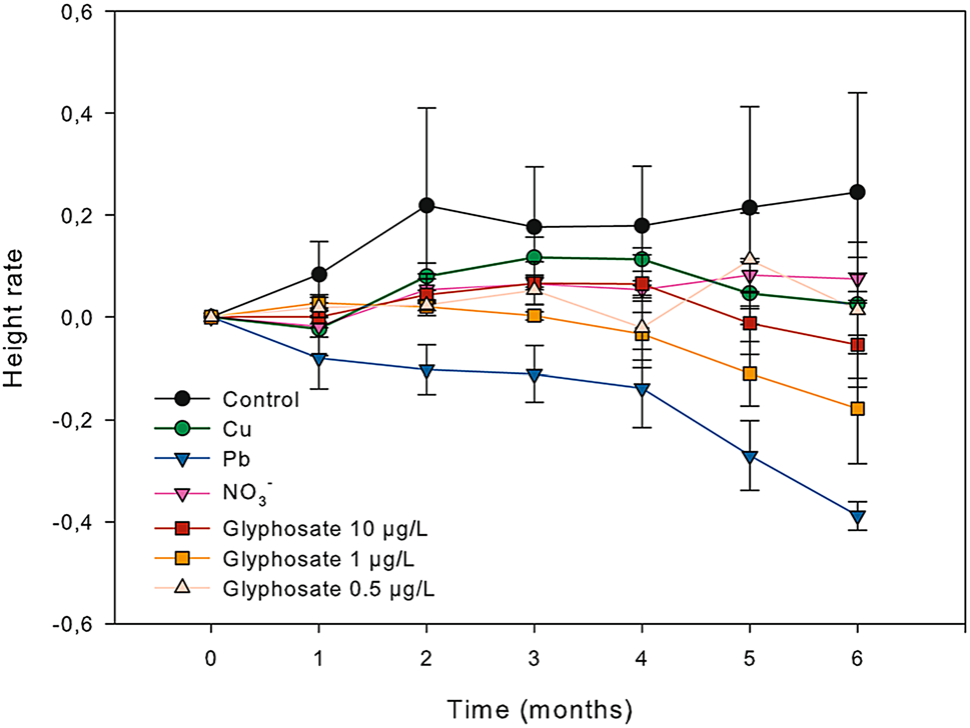
Mean height variation of adult individuals of *Carpodesmia crinita* under control conditions and the different pollutant treatments for 6 months. Height variation is given in parts per unit. Vertical bars represent standard errors.

Similarly, height was more or less constant throughout the experiment for all treatments except for lead, where specimens reduced a 39% initial height after 6 months (Tukey test, *p* < 0.05 for Pb and control, Cu and nitrate treatments; supplementary Table S1 Fig. 3).

**Figure 4:**
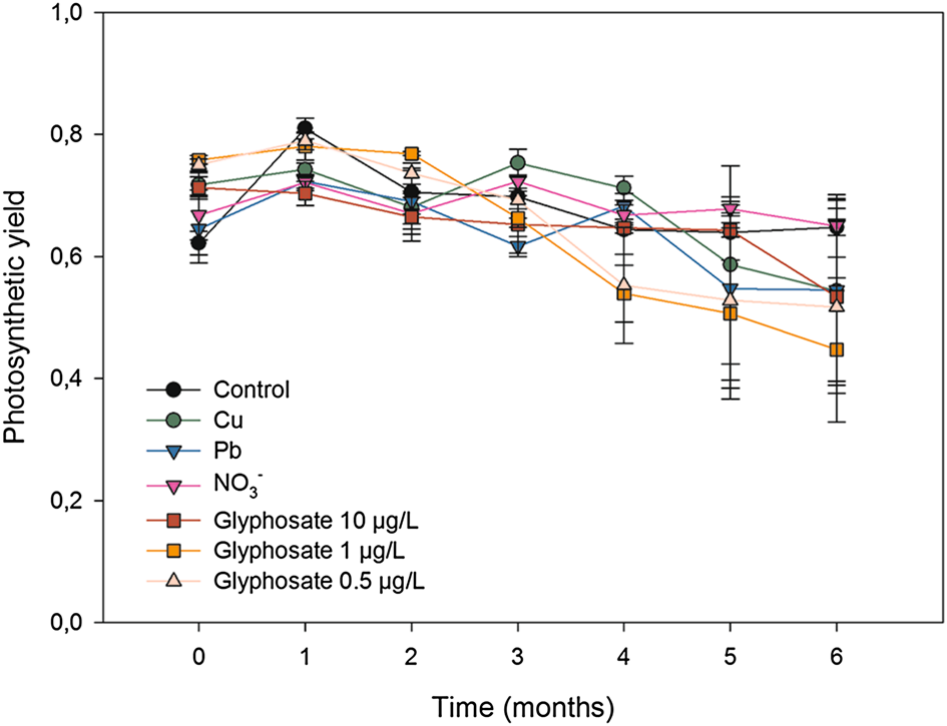
Mean photosynthetic yield (F_v_/F_m_) of adult individuals of *Carpodesmia crinita* under control conditions and the different pollutant treatments for 6 months. Vertical bars represent standard errors.

Mean effective quantum yield (F_v_/F_m_) values did not differ significantly among treatments (χ^2^_6_ = 3.305, *p* = *0.770*; supplementary Table S1; Fig. 4). The main quantum yield values were in the range of, approximately, 0.6 to 0.8 which is considered an optimal range for brown algae^72–75^.

**Figure 5:**
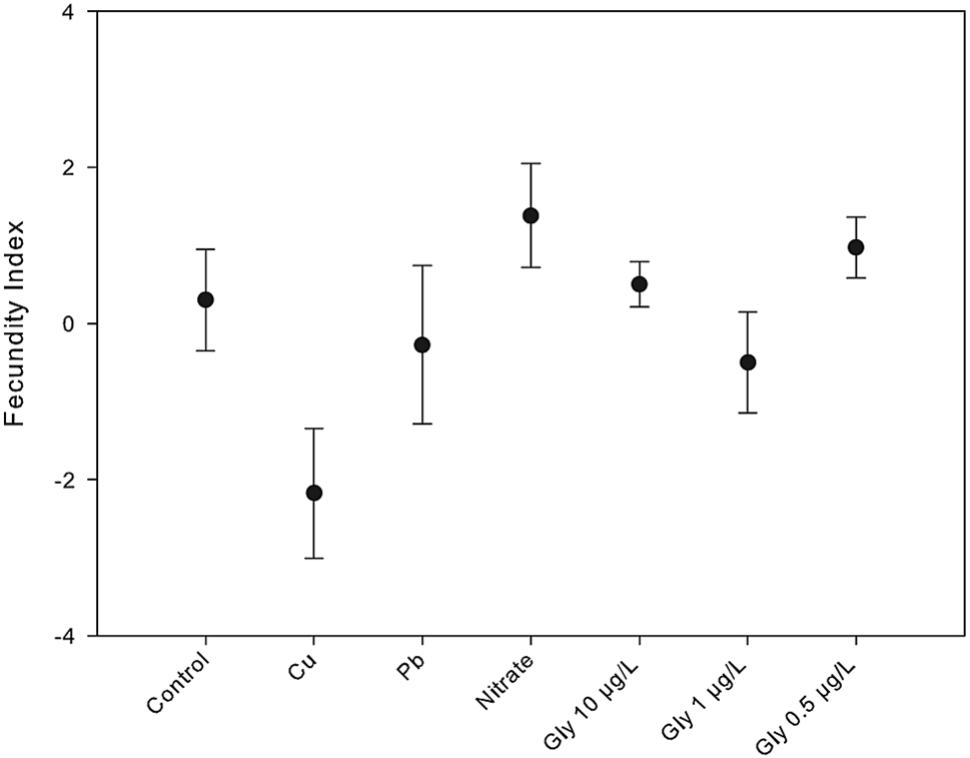
Mean variation of fecundity index of adult individuals of *Carpodesmia crinita* under control conditions and the different pollutant conditions treatments, 6 months after the beginning of the experiment. Vertical bars represent standard errors.

Fecundity level (FL) of adult *C. crinita* specimens at the beginning of the experiment for all treatments was similar, ranging from 1.5 to 2.5 (F_6,21_ = 0.733, *p* = *0.915*). However, by the end of the experiment, the fecundity index had changed significantly among treatments (F_6 ,21_ = 3.087, *p* < *0.05;* supplementary Table S2; Fig. 5). Although there were no differences among the fecundity level of individuals from control, nitrate, Pb, and glyphosates treatments all the specimens from the copper treatment (independent of their initial fecundity level) were sterile (Tukey test, *p* < 0.05 for Cu and glyphosate 0.5 µg/L, and Cu and nitrate).

## Recruit responses

**Figure 6:**
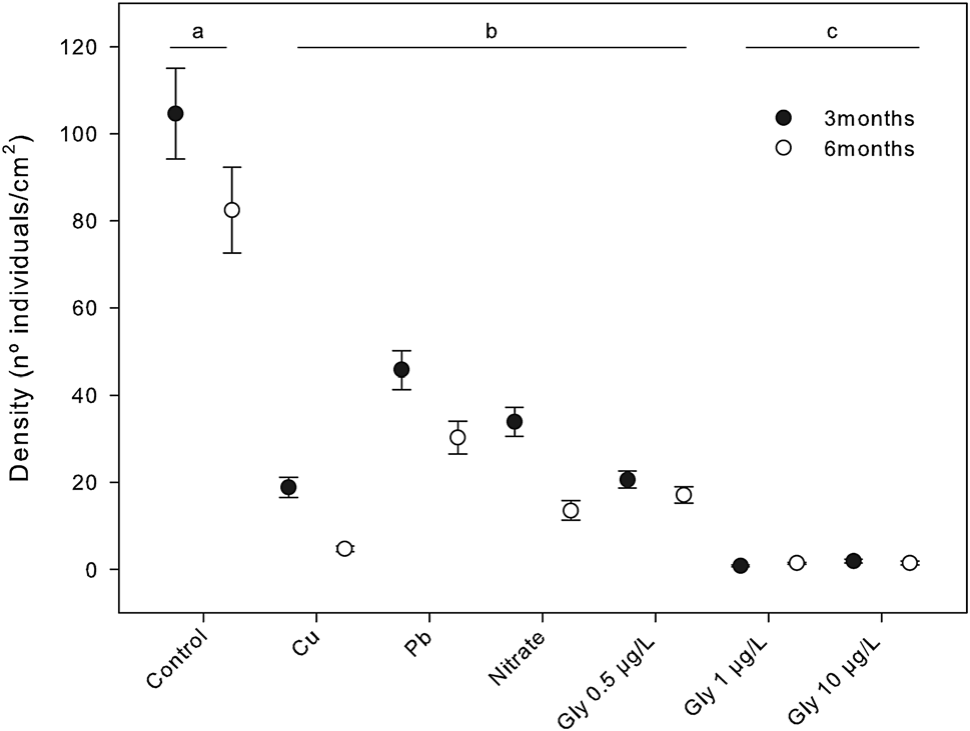
Mean recruit density (number of recruits) of *Carpodesmia crinita* under control conditions and the different pollutant treatments at 3 months and 6 months after the beginning of the experiment. Vertical bars represent standard errors. Horizontal lines (**a**, **b** and **c**) indicate significant differences between treatments.

**Figure 7:**
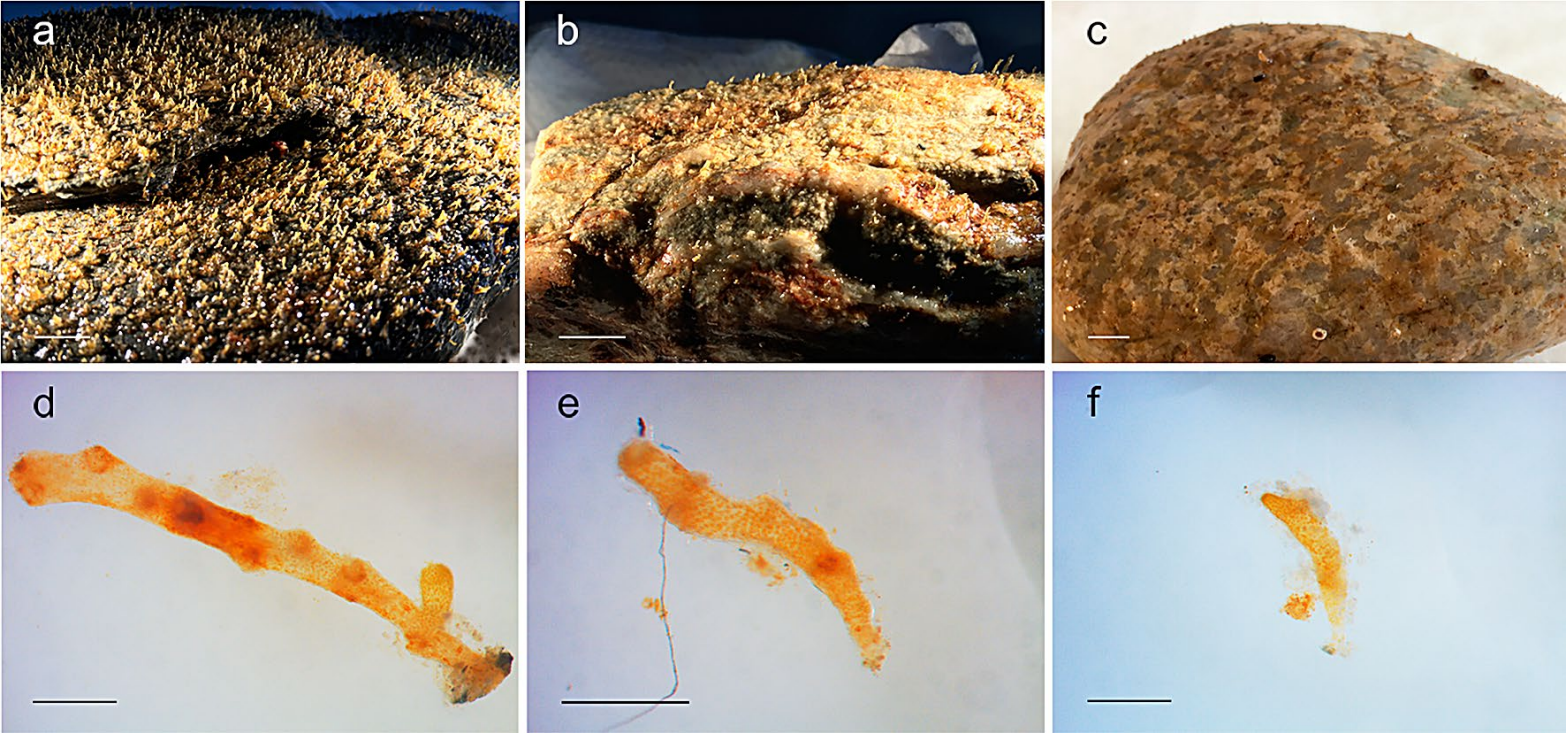
Top: Example of stones with recruits at month 3: (**a**) control, (**b**) nitrate and (**c**) glyphosate 0.5 µg/L. The horizontal scale bars represent 1 cm. Bottom: Recruits of *Carpodesmia crinita* at month 3 under different treatments: (**d**) control, (**e**) nitrate and (**f**) glyphosate 0.5 µg/L. Scale bars represent 0.5 mm.

Recruit density was significantly higher in the control tanks than in the tanks with pollutants at both measuring times, with 104.6 (± 10.36) and 82.5 (± 9.837) mean individuals/cm^2^ (± SE), at month 3 and month 6, respectively (Figs. 6 and 7a–c). The lowest recruit density was found in glyphosate treatments of 1 µg/L and 10 µg/L (ca. 1.5 individuals/cm^2^ at both month 3 and month 6; Tukey test, *p* < *0.001*). However, all the other treatments (Cu, Pb, NO_3_^-^ and 0.5 µg/L glyphosate) also produced lower recruit densities than that of the control treatment, with values at month 6 ranging from 4.733 (± 0.716) to 30.3 (± 3.823) individuals/cm^2^; (Tukey tests, *p* < *0.0001*). The differences in recruit densities among treatments were already evident in month 3, and the same trend was observed at month 6 (χ^2^_6_ = 7785.9, *p* < *0.001*; supplementary Table S2; Figs. 6 and 7a–c).

**Figure 8:**
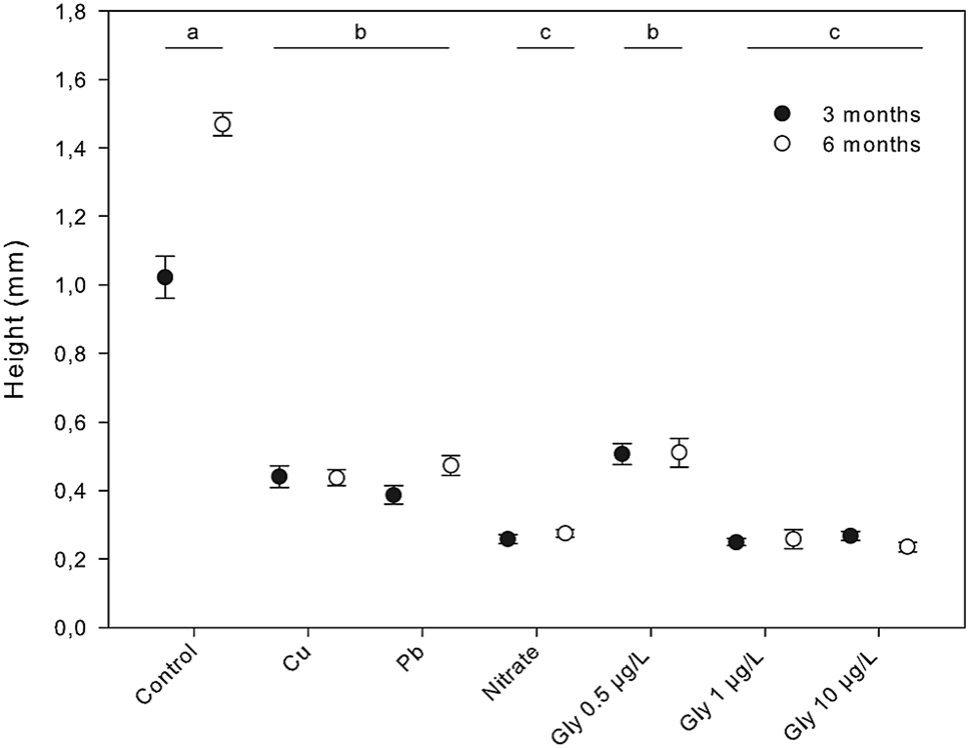
Mean height of recruits of *Carpodesmia crinita* under control conditions and the different pollutant treatments at months 3 (black dots) and 6 (empty dots) after the beginning of the experiment. Vertical bars represent standard errors. Horizontal lines indicate the significant differences between treatments.

Recruits in the control tanks grew to the largest sizes, reaching a mean of 1.468 (± 0.034) mm at months 6 (Tukey tests, *p* < *0.0001* for all comparisons). Recruits treated with Cu, Pb and 0.5 µg/L glyphosate were around four times smaller than the recruits in the control tanks after six months, whereas those under nitrate and 1 and 10 µg/L glyphosate were around eight times smaller. The marked differences in the mean size of recruits under different treatments became evident after 3 months and remained constant until the end of the experiment (F_6,819_ = 178.37, *p* < *0.001*; supplementary Table S2; Figs. 7d–f and 8).

## Discussion

Research into the specific responses of macroalgae to pollutants currently focuses mainly on their capacity to accumulate heavy metals and their use as bioindicators^44,76–78^. Moreover, most studies tend to deal with concentrations that are only found in highly polluted sites, such as areas where domestic, agricultural or industrial sewage discharge occur into coastal waters^50,64,79–81^. This meaning that the effects of toxic compounds are only known for very large concentrations, in places where macroalgal forests have already disappeared.

We have shown, in this study, how subtle pollutant concentrations over a period of six months may have no visible effects on adult populations of macroalgae, but can seriously compromise their mid-term and long-term viability by reducing the fertility of the adults and the survival of recruits. A number of studies have already shown that high levels of eutrophication^36,43,82^, heavy metals^44,83^ and pesticides^50,64^ result in reduction in adult survival, growth and photosynthetic capacity for many species of Fucales, and that this can lead to forests of these species being replaced by simplified systems. However, low to moderate levels of pollution rarely result in an immediate deforestation of fucoid assemblages^43,84^ since fucoids seem to be able to resist a certain degree of pollution. In fact, fucoids can survive in areas with pollution levels two to three times higher than those found at “unpolluted” reference sites^43,44^ which demonstrates that these macroalgae can resist a constant weak pollution source to a certain extent^12,84,85^. However, our results indicate that the lack of observed detrimental effects on adult individuals in well-established populations may well be concealing the real ecological effect – at the population level – of these low concentrations of pollutants.

Based on our results, growth (as changes in weight and height) and the effective yield of *C. crinita* adults seem not to be drastically affected by low but continuous (6 months) pollutant levels. Although most experimental studies on the effects of pollutants on brown algae are short-term experiments, the effects of heavy metals on its growth capacity appear to be species-specific^83^. Some *Fucus* species reduce their growth when exposed to concentrations ranging from 12–50 µg/L of Cu for 10 days and they stop their growth at higher concentration (300 µg/L of Cu)^83^. On the other hand, *Sargassum cymosum* did not change their growth compared to the control treatment when exposed to 10 to 50 µM of Pb for 7 days^86^. Regarding to the effective yield brown algae (*Ascophyllum nodosum* and *Fucus vesiculosus*) were not affected when exposed to Cu levels of 10 µg/L of Cu for 14 days^87^, neither *Sargassum cymosum* under Pb exposition (< 50 µM for 7 days)^86^. However, huge glyphosate levels (882.5 µg/L for 6 days) negatively affect chlorophyll absorbance of *Fucus virsoides*^50^.

We observed a market reduction in survival and growth of recruits at nitrate levels that do not affect adults (Figs. 6 and 8). It has previously been reported that physiological nutrient uptake and saturation mechanisms differ for recruits and adults: a moderate enrichment of nitrates can be beneficial for the growth and development of many adult kelp and fucoid species^88–92^, which accumulate nitrogen intracellularly with non-saturating nutrient uptake kinetics^93,94^, whereas nutrient uptake saturation occurred in recruits^95^. Such physiological differences might be linked to the higher sensibility of recruits to moderate/high nitrate concentrations.

Similarly, studies have shown the capacity of some adult brown algae to resist moderate heavy metal pollution, especially from copper^44,48,96^, but we have shown that even low copper concentrations seriously affect the fertility of adults from the species *C. crinita*, as well as the settlement and growth of recruits. The reasons for the copper impacts are unknown, but macroalgae may respond either by reducing the creation of reproductive structures, which suggests a trade-off between reproduction, growth and survival^97^, or by inhibiting the cell wall formation in zygotes during egg fertilization, thus preventing their normal development^46^. Furthermore, copper also can disrupt zygote germination and reduction in growth and normal development of juveniles of Fucales^45,46,98^ and gametophyte development on kelps^99,100^.

Glyphosate toxicity on marine environments has often been disregarded due to its apparent rapid microbial degradation^101^ and strong absorption by soils and sediments that potentially limit runoff in surface waters^102^. However, recent works show that moderate levels of glyphosate do exist in marine environments^51^ and that its strong capacity to bind with organic matter may help protect it from degradation^103^. The scarce studies dealing with the impacts of glyphosate on marine macroalgae are based only on adult stages and show that high concentrations can strongly affect growth, chlorophyll content and photosynthetic yield^50,64,104,105^. However, at lower concentrations of less than 1 µg/L, commonly found in coastal waters^51,64^, glyphosate did not show any impact on the adults^50,104^. Our experiments confirmed this as there were no signs of impairment on adult specimens of *C. crinita* at glyphosate concentrations of 1 µg/L. Nevertheless, at this concentration, and even at the lower concentration of 0.5 µg/L, we did observe reduced recruitment and increased mortality of recruits. As is the case with terrestrial plants, the toxicity of glyphosate on aquatic photosynthetic macrophytes depends on its ability to inhibit an enzyme implicated in the synthesis of the aromatic amino acids essential for protein synthesis^106,107^ as well as its ability to increase shikimic acid, which is related to a decline in carbon fixation intermediates^108^.

The levels of water pollution were probably not high enough to drive rapid extinctions of algal forests, but in the light of our experiments, we suggest that this pollution probably affected the settlement and growth of new individuals. Since recruitment plays an essential role in ensuring the stability of macroalgal populations, impacts on their recruits could have triggered a gradual decline and even local extinctions of many fucoid populations.

With the implementation of the EU Water Framework Directive (2008/56/EC), water quality has significantly improved in many EU countries^35,41^, but this has not led to a recovery of canopy-forming macroalgal populations^35^. Many water bodies classed as having a “good environmental status” still display chronic but moderate/low levels of pollution^109–111^ which, by preventing the recruitment of new individuals as our results suggest, may explain the fact that natural populations of fucoids are not recovering. This situation is probably not restricted to the Mediterranean Sea, as most marine coastal environments are now polluted to some extent, but we contend that it can be included among the several plausible causes triggering the decline in canopy-forming macroalgae^23^. We must also bear in mind, other factors such as overgrazing, invasive species or increasing temperatures, which may also be significantly involved in the decline of macroalgal populations^112^. Finally, since many areas are subjected to cumulative impacts^112,113^, future experiments that address the cumulative effects of two or more of these stressors will help to elucidate the dynamics of the decline in macroalgae forests. This knowledge needs to be incorporated into future conservation and restoration management in order to ensure the preservation of canopy-forming macroalgal populations and their associated biodiversity and ecosystem services.

Currently we are witnessing a general decrease of fucoid forests in the Mediterranean Sea^26,30–34,59^ which seem not to be able to recover^35^ unless some recovery management actions are implemented^65,114,115^. Thus, the future of Mediterranean fucoid forests must go hand in hand of a sustained decrease in all kind of pollutants in seawater (nutrients, heavy metals and persistent organic pollutants) and management actions oriented to facilitate the recovery of the populations.

## Supplementary information

Supplementary Information 1.

## Data Availability

The datasets analyzed during the current study are available from the corresponding author on reasonable request.
