# Supplementary material for: Differential effects of pollution on adult and recruits of a canopy-forming alga: implications for population viability under low pollutant levels
Source: Sci Rep. 2020 Oct 20;10:17825. doi: 10.1038/s41598-020-73990-5 (PMC7575554; doi:10.1038/s41598-020-73990-5)
Supplement: Supplementary file 1 — Supplementary Information 1. [file 41598_2020_73990_MOESM1_ESM.docx]

**Differential effects of pollution on adult and recruits in a canopy-forming alga: implications for population viability under low pollutant levels**

Sònia de Caralt^1,*^, Jana Verdura^1^, Alba Vergés^1^, Enric Ballesteros^2^, Emma Cebrian^1,2,*^

**Supplementary Table S1.** Results of the different fitted models comparing the different variables (adult weight variation, adult height variation, and optimum quantum yield) through time in relation to the different pollutant treatments (control, nitrate, Cu, Pb and three glyphosate concentrations).

| **Variable** | **Fitted model** | **Factor** | ***df*** | **χ^2^** | ***p*-value** |
| --- | --- | --- | --- | --- | --- |
| **Adult weight**  (%) | LMER | Pollutants | 6 | 7,79 | 0.254 |
| **Adult height**  (%) | LMER | Pollutants | 6 | 24,87 | **<0,001** |
| **Adult yield** (Fv/Fm) | GLMM | Pollutants | 6 | 3,31 | 0,769 |

*For each factor, we report the degrees of freedom and the* χ^2^*- and p-values. The significant values are highlighted in bold.*

**Supplementary Table S2.** Results of the different fitted models comparing the different variables (adult fecundity index, recruit height, and recruit density) through time in relation to the different pollutant treatments (control, nitrate, Cu, Pb and three glyphosate concentrations).

| **Variable** | **Fitted model** | | **Factor** | ***df*** | ***F*-value** | ***p*-value** |
| --- | --- | --- | --- | --- | --- | --- |
| **Adult fecundity index** | | LM | Pollutants | 6 | 3,087 | **0,0251** |
| (FI= FL_f_ – FL_i_) | |  |  |  |  |  |
|  | |  |  |  |  |  |
| **Recruit density** | | GLM | Pollutants | 6 | 12200,8 | **<0,001** |
| (num ind/cm^2^) | |  | Time | 1 | 454,2 | **<0,001** |
|  | |  | Pollutants * Time | 6 | 277,8 | **<0,001** |
|  | |  |  |  |  |  |
| **Recruit height** | | LM | Pollutants | 6 | 282,79 | **<0,001** |
| (mm^2^) | |  | Time | 1 | 22,636 | **<0,001** |
|  | |  | Pollutants * Time | 6 | 16,152 | **<0,001** |

*For each factor, we report the degrees of freedom and the F- and p-values. The significant values are highlighted in bold.*
